# Identification of novel immune regulators of tumor growth using RIPPS screening in vivo

**DOI:** 10.1186/2051-1426-3-S2-P66

**Published:** 2015-11-04

**Authors:** Tom Brennan, David Bellovin, Jacqueline de la Torre, Nebiyu Wondyfraw, Servando Palencia, Kevin Hestir, Ernestine Lee

**Affiliations:** 1 FivePrime Therapeutics Inc., San Francisco, CA, USA

## 

Identification of novel targets in cancer immunotherapy is needed to address the significant number of patients that either do not respond to current therapies or encounter unacceptable toxicities. The discovery of such targets, including novel checkpoint regulators and the counter-receptors for previously “orphan” checkpoints, has been limited by a lack of a comprehensive collection of proteins suitable for functional screening and methods for assessing their function in high-throughput.

We have generated a comprehensive library of substantially all human extracellular proteins, encompassing nearly every target for protein therapeutics. Our library contains more than 5700 proteins, including secreted protein ligands and the extracellular domains of membrane-bound receptors in soluble forms. The library proteins represent therapeutic targets and in some cases may act as therapeutics themselves. A portion of this library we call the immunome contains ~500 proteins with structural features characteristic of immune-activators and checkpoints that we selected.

RIPPS^SM^ technology is a robust method whereby FivePrime's library of soluble secreted proteins can be tested *in vivo* in virtually any disease model. Each cDNA representing a unique protein is administered to a cohort of mice and results in high circulating levels of the encoded protein. RIPPS^SM^ also allows us to rapidly confirm activity identified by other *in vitro* screening approaches. Here, we have exploited RIPPS^SM^ technology to screen for new immuno-oncology therapeutics and targets for therapeutic development. As positive controls we performed RIPPS^SM^ on CT-26 tumor-bearing mice using known agonists and antagonists of the immune response, which resulted in decreased or increased tumor growth, respectively. Subsequently, we screened over 250 immunome proteins by RIPPS^SM^ in the CT-26 tumor model and have identified proteins that enhance and inhibit tumor growth and display changes in TIL (tumor-infiltrating lymphocyte) profiles. In addition, the immunome was screened *in vitro* for the ability to modulate anti-CD3-stimulated human T cell proliferation[1]. This screen identified a number of known checkpoint regulators, which validated the assay, and identified numerous novel co-inhibitory proteins – the majority of which have no published link to immune cell regulation. Proteins identified in the *in vitro* screens have been validated using RIPPS^SM^ and other *in vivo* methods. These data demonstrate the power of our discovery platform to discover and validate novel therapeutic targets and protein therapeutics for immuno-oncology.
